# Software platform virtualization in chemistry research and university teaching

**DOI:** 10.1186/1758-2946-1-18

**Published:** 2009-11-16

**Authors:** Tobias Kind, Tim Leamy, Julie A Leary, Oliver Fiehn

**Affiliations:** 1 UC Davis Genome Center, Metabolomics, 451 Health Sci Drive, Davis, California 95616 USA; 2 UC Davis IET, Academic Technology Services, Surge II, Hutchison Drive, Davis, California 95616 USA; 3 UC Davis, Department of Molecular and Cellular Biology, 1 Shields Rd, Davis, California 95616 USA

**Keywords:** Virtual Machine, Virtual Machine Monitor, Guest Operating System, Antivirus Software, Server Consolidation

## Abstract

**Background:**

Modern chemistry laboratories operate with a wide range of software applications under different operating systems, such as Windows, LINUX or Mac OS X. Instead of installing software on different computers it is possible to install those applications on a single computer using Virtual Machine software. Software platform virtualization allows a single guest operating system to execute multiple other operating systems on the same computer. We apply and discuss the use of virtual machines in chemistry research and teaching laboratories.

**Results:**

Virtual machines are commonly used for cheminformatics software development and testing. Benchmarking multiple chemistry software packages we have confirmed that the computational speed penalty for using virtual machines is low and around 5% to 10%. Software virtualization in a teaching environment allows faster deployment and easy use of commercial and open source software in hands-on computer teaching labs.

**Conclusion:**

Software virtualization in chemistry, mass spectrometry and cheminformatics is needed for software testing and development of software for different operating systems. In order to obtain maximum performance the virtualization software should be multi-core enabled and allow the use of multiprocessor configurations in the virtual machine environment. Server consolidation, by running multiple tasks and operating systems on a single physical machine, can lead to lower maintenance and hardware costs especially in small research labs. The use of virtual machines can prevent software virus infections and security breaches when used as a sandbox system for internet access and software testing. Complex software setups can be created with virtual machines and are easily deployed later to multiple computers for hands-on teaching classes. We discuss the popularity of bioinformatics compared to cheminformatics as well as the missing cheminformatics education at universities worldwide.

**Electronic supplementary material:**

The online version of this article (doi:10.1186/1758-2946-1-18) contains supplementary material, which is available to authorized users.

## Introduction

**Figure 1:**
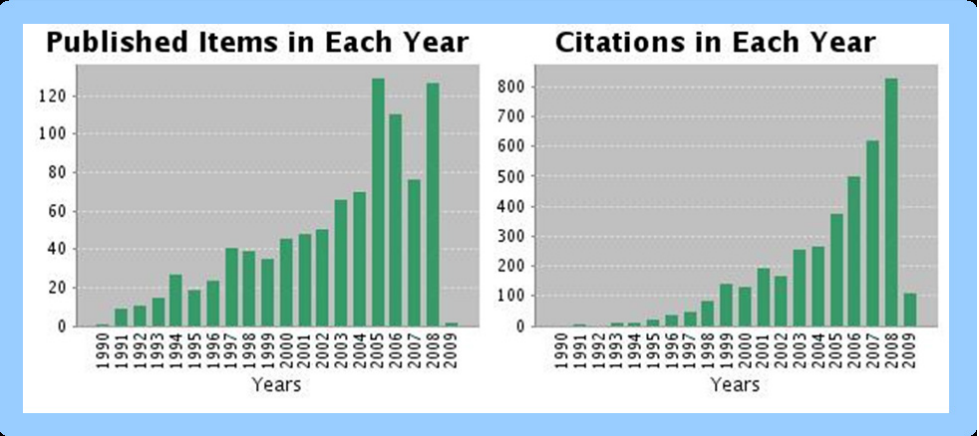
**Number of scientific papers and citations about virtualization and virtual machines**. Source ISI Web of Science January 2009.

"Virtual machines have finally arrived. Dismissed for a number of years as merely academic curiosities, they are now seen as cost-effective techniques for organizing computer systems resources to provide extraordinary system flexibility and support for certain unique applications." This statement from one of the pioneers of virtualization (Goldberg 1974 [1]) is equally true 35 years later and the hype generated by the computer science community and software companies require special attention especially in chemistry and life sciences because virtual machines have undoubtedly and truly arrived. More than 50 million hits in Google and around 1000 scientific papers (see Figure 1) show the significance of software virtualization. This technology allowed companies like VMWare to reach the top five of all software companies within 10 years reaching a market capitalization of almost 20 billion dollars in 2008. Little is known about applications and use of software virtualization in life sciences and especially chemistry. In this paper we discuss basic parts of the technology; investigate the performance of chemistry software and discuss advantages and disadvantages of virtual machine applications in chemistry, cheminformatics [2], chemometrics [3], mass spectrometry laboratories and university teaching classes.

### Software platform virtualization

**Figure 2:**
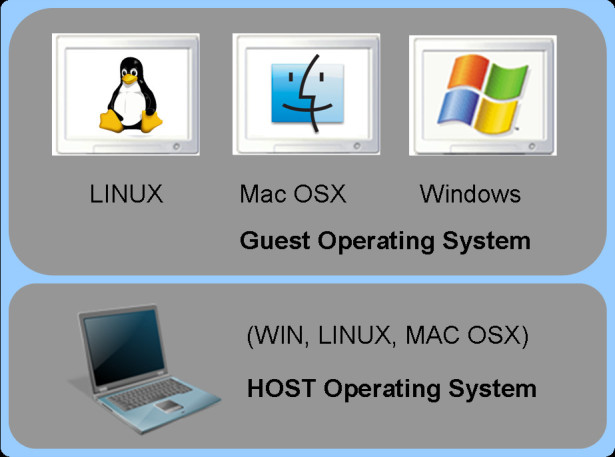
**The virtual machine software installed on a host operating system allows the use of different operating systems on a single computer system**. A Macintosh system could run native Windows or LINUX software or even multiple instances of the same operating system. All virtual machines can communicate with each other and are allowed to use all hardware computer resources such as graphic cards, DVD drives and USB ports. (Logo sources: Wikipedia, TUX mascot: Larry Ewing).

Software platform virtualization [4] (system virtual machine) allows a single host operating system to execute multiple guest operating systems on the same computer without rebooting. For example a computer running Microsoft Windows can run an independent LINUX operating system in a separate window or vice versa. Also a computer running Mac OS X can use virtualization programs to independently run operating systems like LINUX or Microsoft Windows in a separate window with the same behavior like a native Macintosh application (see Figure 2). The term application virtualization [5] (process virtual machine) refers to products such as the JAVA virtual machine, as a result JAVA applications can be run on different operating systems (write once, run anywhere). One reason to use platform virtualization is that programs compiled for a specific operating system can only run under the same operating system. Therefore software programs which are compiled for Microsoft Windows only run under Microsoft Windows, unless any other bytecode emulator or translation layer such as WINE is used [6]. For software exchange between the host and guest operating system a shared folder can be used or software can be moved with the mouse via drag-and-drop or additional network connections. Other operational modes include hypervisor server virtualization (Hyper-V) [7] or hardware virtualization concepts for better performance, such as Intel Virtualization Technology (Intel-VT) and AMD Virtualization (AMD-V).

### Virtual machines exist for Windows, LINUX and MAC OS X

**Figure 3:**
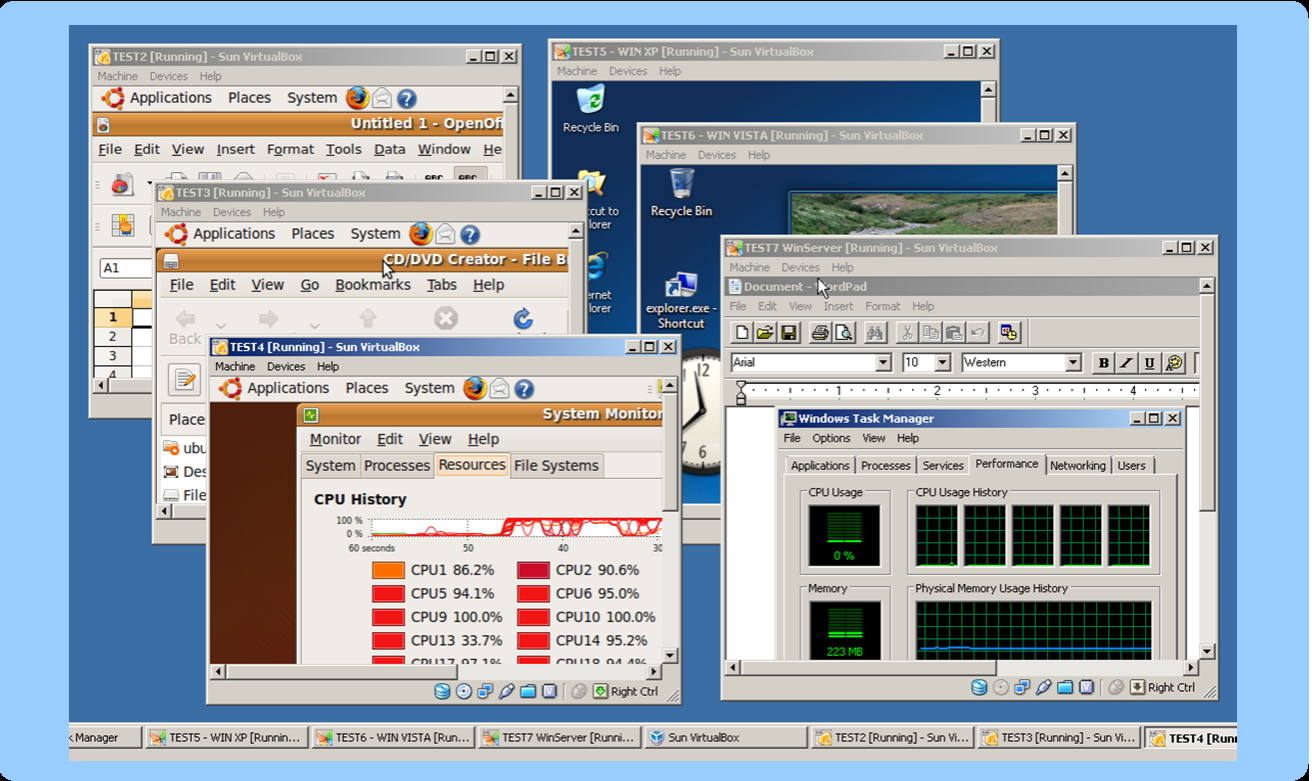
**A Windows Vista host using Sun's VirtualBox runs three UBUNTU Linuxes, one WIN XP, one Windows VISTA and one Windows Server guest operating system simultaneously**. The hardware is an Intel Nehalem Core i7 950 quad core CPU (3 GHz) with 12 GByte RAM and 4 hard disks in RAID10. The system virtualizes a total number of 41 CPUs.

Around fifty commercial or free open-source solutions are currently available [8]. We discuss some of the commonly used desktop virtual machines (VMs) as shown in Table 1. For the Windows platform the VMware Workstation for Windows and the free Microsoft Virtual PC are among the most popular programs. For LINUX as host the VMware Workstation for LINUX and XEN are commonly used. For Mac OS X as host VMware's Fusion, Parallels Desktop and the open source Sun Microsystems' VirtualBox are available. Although virtually every operating system can be used both as host and as guest, running Mac OS X (Tiger) as a guest operating system in a virtual machine is currently prohibited by the software maker Apple Inc. Only the server operating system Mac OS X Server 10.5 (Leopard) allows virtualization, but only if original Apple hardware is used. In case different virtual machine software solutions are installed, it is sometimes required to convert virtual disk images to allow the use with other virtual machines [9]. Such converter software tools can convert VMware, Microsoft Virtual PC, Citrix XenServe, Virtual Iron and even backup solution images from Acronis True Image or Symantec Ghost [10]. Figure 3 shows six different guest operating systems running simultaneously on a Windows Vista host computer workstation using Sun's VirtualBox.

**Table 1:** List of common desktop virtual machines for Windows, LINUX and Mac OS X operating systems.

Host OS	Virtualization Software	WINDOWS as Guest OS	LINUX as Guest OS	Mac OS X as Guest OS
Windows OS				
	VMware Workstation	Yes	yes	no
	Microsoft Virtual PC	Yes	yes	no
	SUN Virtual BOX	Yes	yes	no
LINUX OS				
	VMWare	Yes	yes	no
	Citrix XEN	Yes	yes	no
	Virtual Iron	Yes	yes	no
MAC OS				
	VMWare Fusion	Yes	yes	yes*
	Parallels Server	Yes	yes	yes*

Mac OS X can in principle run on any host, but it is not officially supported. A star (*) denotes license issues (January 2009).

### Server consolidation by using virtual machine software

**Figure 4:**
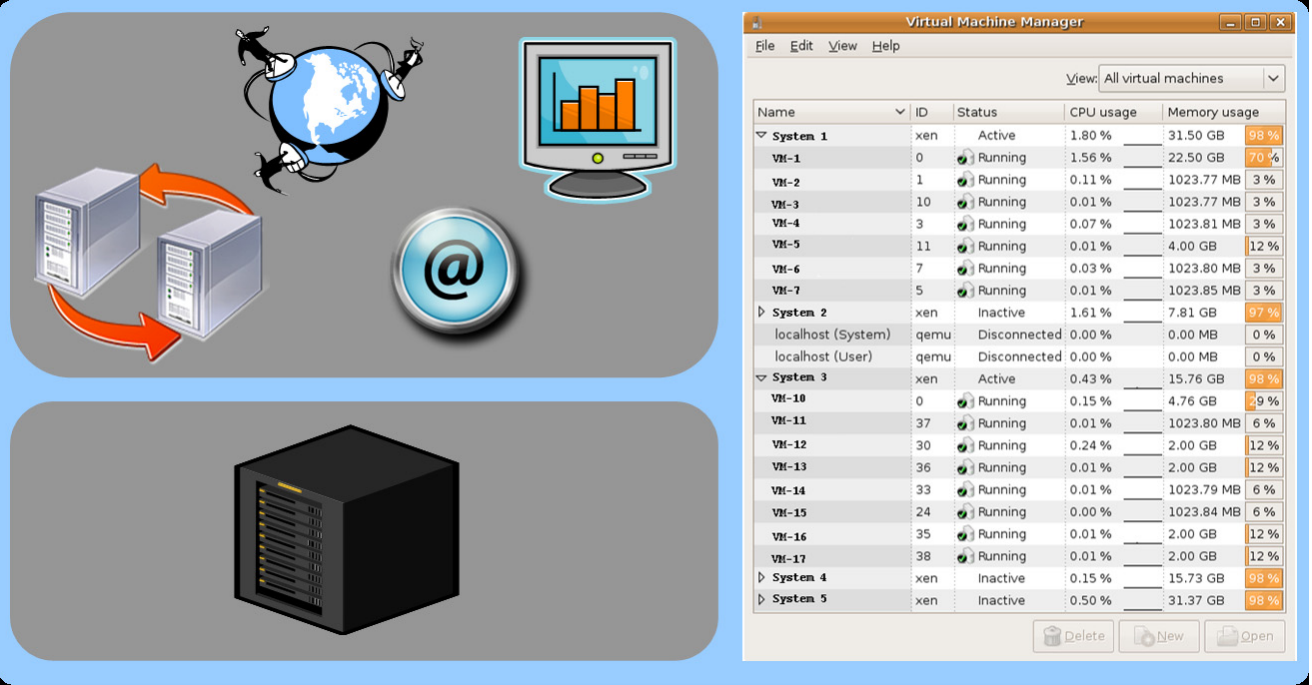
**Server consolidation: A single powerful computer runs multiple virtual machines and serves as compute server, backup server and web server**. Such a setup improves maintenance efficiency and reduces hardware costs. The right picture shows a production server with a XEN Virtual Machine Monitor and 17 independent running systems (Actual VM names were replaced; Picture source: Zhi-Wei Lu; UC Davis Genome Center Bioinformatics Core).

The term server consolidation refers to the concept of replacing a number of older computers with a single multi-core or multi-CPU system [11]. For example eight computers each having oneGByte memory and one single CPU, could be replaced by a single powerful computer with a dual quad-core-CPU setup and a total of 8 GByte memory. The initial aim of server consolidation is to save energy as well as hardware and maintenance costs. Energy can be saved by using newly designed processors with a better performance per Watt ratio. Operating and management costs can be saved because the systems administrator only has to deal with a single physical computer instead of multiple computers [12]. By using hypervisor server virtualization software a series of different operating systems can be installed into independent virtual machines. The right picture in Figure 4 shows a XEN Hypervisor (Virtual Machine Manager) with 17 independent virtual machines across two physical servers. The hardware setup for all virtual machine installations is the same, resulting in fewer problems with different software drivers for components such as graphic cards, network adapters and hard disks.

### Software virtualization and multiple operating systems in chemistry

The literature coverage of virtualization concepts in chemistry is extremely sparse. One paper discusses the use of virtualization on supercomputers for molecular dynamics calculations utilizing more than 1550 processors [13]. Another chemistry related publication discusses the use of virtualization software in the pharmaceutical industry for virtual screening and lead optimization in a grid-like environment [14]. The use of VMs among software designers may be higher and is not fully reported in the peer-reviewed literature. The biggest advantage of installing virtual machine software is the ability to run applications from different operating systems on a single computer. Another advantage is to test software without the need of installing software into a working production environment. A similar approach is used with live-CDs that contain a bootable operating system with pre-installed software, such as the free Vigyaan electronic workbench for bioinformatics, computational biology and computational chemistry [15]. Such live-CDs can be easily mounted to virtual machines without needing to reboot the original production system. Furthermore, the Microsoft Windows operating system is known to slow down after installation of hundreds of software tools. That can be prevented by installing software into a virtual machine. We will investigate possible speed penalties during the use of virtual machines with a series of scientific benchmarks (see Table 2). For software development purposes software virtualization is used to compile native solutions and test software on different operating systems. Besides that, different software versions can show incompatibilities with data files created with different software versions. In such a case old software must be un-installed and new software must be re-installed. In case of platform virtualization, every new software version is installed into a single independent operating system. Every system change can be completely reversed with the included differential snap-shot system. In a university teaching environment platform virtualization is a fast way to deploy copies of the same installation file to multiple computers in a classroom.

**Table 2:** List of system statistics and micro-benchmarks comparing an original Windows XP performance and Windows XP inside a virtual machine (Guest OS).

ID	Task	WINDOWS XP Host	WINDOWS XP Guest VM	of Guest VM
System benchmarks				
1	Operating system start time	2 min	1 min	50% less time
2	Size of windows system folder	6.95 GByte	3.01 GByte	57% less space
3	RAM memory requirement (IDLE)	760 MByte	150 MByte	80% less RAM
4	Average hard disk transfer rate	180 MByte/sec	127 MByte/sec	70%
**Single CPU core benchmarks**				
5	NIST SciMark 2.0a (JAVA 1.6 Server)	score of 661	score of 621	94%
6	Molgen Demo - count all23862255 isomers of C12H12	42.23 sec	46.20 sec	91%
7	CDK Descriptor GUI -- Kier & Hall SMARTS for all C8H16O2 isomers	100 sec	95 sec	95%
8	Seven Golden Rules -- generate all 28008691 formulas below 1000 Da	42 sec	42 sec	100%
**Dual CPU core benchmarks**				
9	ChemAxon Marvin - calculate all stereoisomers of C8H16O2	21 sec	42 sec	50%
10	MZMine2 -- chromatographic alignment of LC-MS runs	70 sec	130 sec	54%

Compared are an aged 2 year old Windows XP (Host OS) and a clean installed Windows XP system (Guest OS) on Microsoft Virtual PC 2007 on a Dual Opteron 254 (2.8 GHz).

### Mass spectrometry and cheminformatics software

Modern mass spectrometers produce data at such high rates that wet lab work is almost minimized to 20% of the relative project time and 80% of the time is spent with computerized data evaluations and investigation of raw data. Due to the strong interconnection of molecular spectra and molecular structures not only mass spectrometry software is used during structure elucidation, but a diverse set of programs for handling structures and for the computation of molecular properties [16]. Such software includes tools for structure elucidation and mass spectrum interpretation, chromatographic peak deconvolution software, biomarker identification and alignment software, software for molecular formula determinations, software for mass spectral library search and chemical structure and descriptor generation (see Table 3). We will discuss the advantages of software platform virtualization in research and university teaching and show some practical applications while focusing on mass spectrometry and cheminformatics applications.

**Table 3:** Cheminformatics and mass spectrometry software course as part of an experimental mass spectrometry class, some of the software was deployed using WIN XP virtual machines in the computer laboratory.

General course	Topics covered
General Introduction	Fighting computer illiteracy -- bits, bytes, CPUs
	Regular expressions as emergency helpers
	Structures -- resonance forms, stereoisomers, tautomers
	Mass spectrometry publications via Yahoo Pipes
**Mass spectral and molecular data handling**	Mass spectral data formats and conversion of mass spectra
	Open exchange formats for mass spectra (mzData, mzXML, JCAMP-DX, netCDF)
	Structure handling software and structure conversion (SMILES/SMARTS, SDF/MOL, InChI/InChIKey, PDB, CML)
	Chemical structure handling (Instant-JChem, BioClipse)
**Mass spectral and molecular database search**	Mass spectral databases (EI, ESI, APCI) and search algorithms (PBM, dot product, mass spectral trees) and library conversion
	Proteomics data analysis (database search, de-novo sequencing, hybrid methods)
	Molecule search (exact search, substructure search, similarity search, Markush search)
	Databases (PubChem, SciFinder, Beilstein, BlueObelisk)
**Mass Spectrometry Tools & Concepts**	Resolving power, mass accuracy, isotopic pattern, charge states, charge state deconvolution
	Molecular formula space of small molecules
	Isotopic abundances as orthogonal filter for elemental compositions
	Molecular Isomer Generators, substructure predictions, simulation of mass spectra
**Concepts for GC-MS**	Automatic peak detection
	Peak picking and mass spectral deconvolution
	Comprehensive GCxGC-TOF-MS
**Concepts for LC-MS**	Deconvolution and evaluation of LC-MS data
	Adduct removal and detection during ESI-LC-MS
	Seven Golden Rules for generation of possible molecular formulas
	Structural isomer lookup example in ChemSpider
**Prediction and simulation of mass spectra**	Dendral - Artificial intelligence and mass spectrometry
	Prediction of the isomer substructures from a given mass spectrum
	Simulation of mass spectra from given isomer structures

## Experimental

### Virtual machine hardware for benchmarks and teaching labs

**Figure 5:**
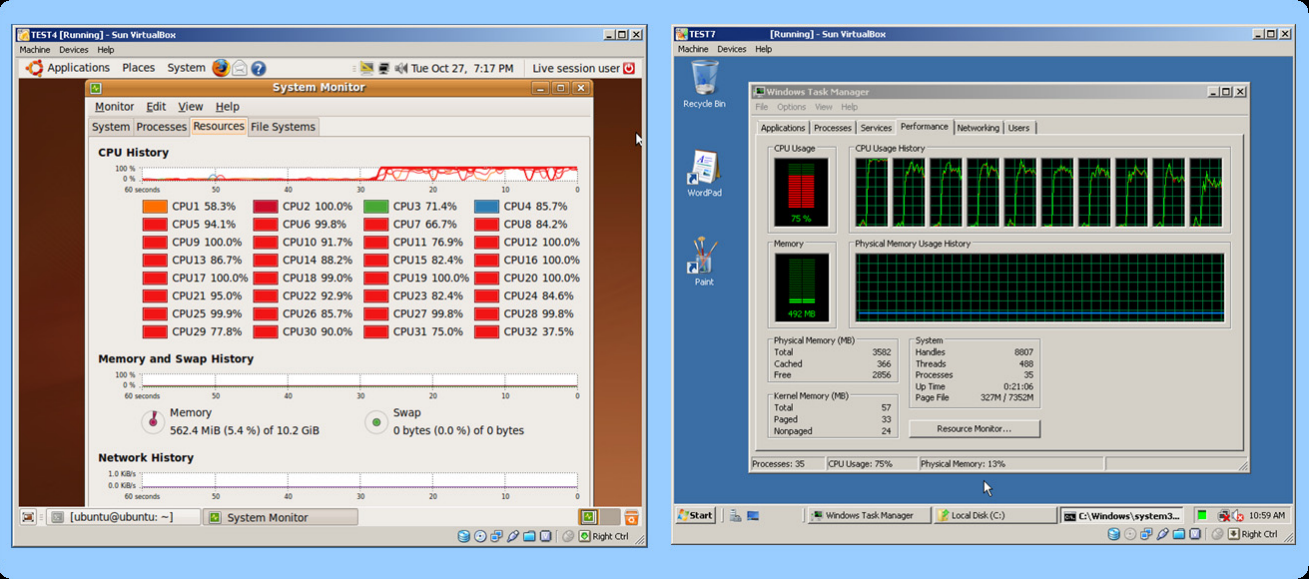
**The Windows Vista Ultimate host with Sun's VirtualBox virtualizes an Ubuntu Linux system with 32 CPU threads (left side) and a Windows Server system with 10 CPU threads (right side)**. The guest hardware is a quad core Nehalem Core i7 950 CPU with only 8 threads. Both guest systems work without problem, but fully exhaust all underlying hardware resources when all parallel threads are in use.

The test system (host computer) for benchmarks was a Dual Opteron 254 (2.8 GHz) with an ARECA-1120 Raid 6 array using WD Raptor hard disks equipped with 2.8 GByte RAM running a 32-bit Windows XP. The forty-one computers for the classroom teaching consisted of: Dell Optiplex GX745 computers with 2.4 GHz Intel Core 2 Duo processors, 4 GB of RAM, and a 160 GB hard drive. These computers had the freely available Microsoft Virtual PC 2007 installed and were configured to allow students to logon using their UC Davis computer accounts. The additional test hardware shown in Figure 3 and Figure 5 was an Intel Core i7 (3 GHz) quad-core system equipped with four hard disks in RAID10 (mirrored stripe) and 12 GByte memory. The hardware shown in Figure 4 was a dual Intel Xeon E5430 quadcore CPU (2.66 GHz) system with 32 GByte RAM and 24 × 1TB Seagate Barracuda ES.2 hard disks using an RAID6 SAS hardware controller.

### Software installation for virtual machines

The freely available Microsoft Virtual PC 2007 (Version 6.0.156.0) was downloaded from Microsoft and was used for all benchmarks and teaching VMs. Memory settings for the virtual machine were set to one GByte RAM. A virtual machine for Microsoft Virtual PC 2007 was created using Microsoft XP (service pack 3) under a university volume licensing agreement. Multiple mass spectrometry and cheminformatics related software packages were downloaded from their original download websites [16] and installed into the virtual machine by simply drag-and-drop copy from one window to another or direct download from within the virtual machine. For all packages an appropriate software license was obtained. Multiple topics were covered in the teaching sessions (see Table 3) but a discussion of each single package goes beyond the scope of this paper. A free teaching license was obtained for the ChemAxon Marvin [17] and ChemAxon Instant-JChem package. The freely available Instant-JChem and the open source BioClipse package [18] were used for GUI driven molecule and spectral handling. Software settings for appliances shown in Figure 3 and Figure 5 included a Windows Vista Ultimate 64-bit operating system and the freely available Sun VirtualBox 3.0 as virtual machine software.

### Benchmark software selection for virtual machine testing

The NIST SciMark 2.0 program [19] was selected because it has cross-platform capabilities and is freely available. Furthermore the five computational routines including Fast Fourier Transforms (FFT), Jacobi Successive Over-relaxation (SOR), Monte Carlo integration, Sparse matrix multiply dense LU matrix factorization represent a fair mix of scientific computing problems. The application is only single-threaded and was obtained from [20]. The benchmark was run with the Sun JAVA 1.6 server compiler in off-line mode. The Molgen program is a molecular isomer generator which requires a molecular formula as input and subsequently counts or generates all possible structural isomers [21]. It was included because the process of isomer generation is of importance in analytical chemistry. The demo version 3.5 (single threaded) was downloaded from [22] and all isomers for C_12_H_12_ were counted. The free CDK Descriptor GUI (v0.94) [23] is a software for molecular descriptor calculation and is based on the open source chemistry development kit [24]. The Kier and Hall descriptors (electrotopological E-state state indices) [25] were used on a dataset of all 13190 C_8_H_16_O_2_ isomers generated with the SMOG2 isomer generator software [26]. The high-speed molecular formula calculator HR2 was downloaded from [27] and performed a generation of all 28,008,691 elemental compositions including the elements (C:1-78 H:1-126 N:0-20 O:0-27 P:0-9 S:0-14) below 1000 Da according to the Seven Golden Rules [28]. The test conditions are below the maximum element restrictions for this mass range and were genuinely chosen for performance measurements. The software Marvin 5.1.4 was downloaded from [17] and used for the generation of all tetrahedral and double bond stereoisomers of C_8_H_16_O_2_. The command was invoked via the command line cxcalc command by supplying a file with all SMOG2 structural isomers. The Marvin software is multi-threaded, hence can make use of multiple CPUs. As a final test the multi-threaded and compute cluster-ready MZmine software was used. It is a package for LC-MS chromatogram alignment [29] and the free mzmine2 (beta 1.92) software was downloaded from [30]. The test set was downloaded from [31] and is based on a metabolic profiling study [32]. All samples were included and batch processed using a zoom scan filter, three steps peak detector (local maxima mass detection, score connector chromatogram construction, Savitzky-Golay peak recognition) and alignment join aligner. Other software licenses were either purchased or obtained through university software licensing. All benchmarks were run three times and the average times were reported using the timethis command from the Windows 2000 Resource Kit Tools.

### Popularity comparison of bioinformatics versus cheminformatics

For the comparison of the popularity of cheminformatics versus bioinformatics a web site specific search was performed on all 325 university websites (US) with an associated research chemistry faculty. A domain specific Google search was used to obtain information how often the words "cheminformatics", "chemoinformatics" and "bioinformatics" occur on a single university website. For example *cheminformatics site:berkeley.edu* returned 93 hits on Google, meaning the word occurred 93 times in HTML websites, in PDF and EXCEL sheets across the whole UC Berkeley website. If zero hits were returned the word would not occur on the specific university website. A JAVA program using the Google web search API was implemented to perform an automated analysis of the several thousand searches. The hit counts for all universities are discussed in the result section. All results are freely available in an EXCEL sheet from the Additional file 1.

## Results

### Installation and micro benchmarks in a research environment

The initial installation size of the virtual machine file with Windows XP SP3 32-bit (guest OS) was 4 GByte. After the installation of multiple software packages the VM file grew to eight GByte, even though the total software installation size was only around 300 MBytes. One of the reasons may be the included swap file or NTFS file system fragmentation and folder compression in the guest OS. It has been reported that the minimum size of a Windows XP system can be as small as 700 MBytes [33]. There are in general two different disk types: fixed disk and dynamic disk systems [34]. A fixed disk has a constant file size. Dynamic disks in a virtual machine can grow up to the maximum available size on the host operating system. During this setup a dynamic disk was selected to leave enough room for installed programs and allow a flexible disk size management. After installation the virtual machine drive has to be defragmented and precompacted with a special precompactor program which zeros out all free space. Additionally the virtual machine has to be stopped and an external defragmentation has to be applied. General system benchmarks can be found in Table 2. The memory footprint of the WIN XP virtual machine guest OS was relatively small with 100 MByte RAM and after installation of the Sophos-Antivirus software the memory allocation grew to 150 MBytes. As comparison a two year old Windows XP system with hundreds of different software tools installed requires up to 500 MByte memory in idle mode (doing nothing) due to multiple drivers and resident programs. The start and save time for a virtual image OS are quite fast. Saving the virtual machine state takes around 30 seconds and restoring the saved virtual machine takes only 5 seconds. This short save time is due to the three times faster average transfer rate of the RAID 6 file system on the host computer compared to a common desktop hard disk. The times for the compute intensive single CPU core benchmarks are listed in Table 2 and it can be seen that the speed penalty for running programs inside a virtual machine usually varies between 5% and 10%. To line out the importance of a fast disk system and multi-core and multi-CPU capabilities of programs and virtualization software also dual core CPU applications were included. The Microsoft Virtual PC does not support symmetric multi-processing (SMP), hence supports only one CPU. The penalty for running in a virtual single CPU virtual environment is 50% lower speed (Table 2).

### Installation and use of VMs in a teaching environment

The virtual image with all the required mass spectrometry and cheminformatics software was deployed to each computer station. Due to the size of the virtual image (eight GByte) it was copied to each PC during off hours. A real-time deployment over network services to multiple computers was impossible. Students and teacher would login into the original computer workstation using their campus Kerberos authentication system. The Virtual PC is then started using the start menu without any additional certification. The set of pre-installed programs is then used for learning structure handling techniques and mass spectrometry data handling approaches including molecular formula generators, charge state deconvolution, isotopic pattern generators, mass spectral database search, tools for mass spectral interpretation and simulation, gas chromatography and liquid chromatography (GC-MS and LC-MS) deconvolution software and tools for mass spectral interpretation and simulation (see Table 3). Additionally the course includes structure handling approaches as well as the exploration of different file formats, structure search techniques and structural isomer generators. The whole set of teaching slides including all software references can be freely downloaded from source [35]. All virtual machines are identical allowing a synchronized working from the instructor's large screen together with all students, who basically follow the instructions and take part in discussions.

## Discussion

### Application of VMs in research and system benchmarks

The micro benchmarks were performed to validate the use under heavy computational tasks. The benchmark programs were selected according to frequent use in cheminformatics and mass spectrometry laboratories. The Microsoft Virtual PC only utilizes one single CPU. Therefore all results are based on single CPU speed instead of utilizing the dual core capabilities. The MS Server version and VMWare virtual machine also allow multiple CPU setups but were not used for comparison. The fastest mode in a virtual machine is the direct execution mode [36] where the machine code runs without interaction from the virtual machine at almost the same native speed. Certain CPU specific commands are prevented from running within the virtual machine or they generate a CPU exception and therefore the virtual machine is needed to emulate such a machine code via binary translation and is much slower. That also explains the very small CPU based speed penalty (virtual machine overhead) for running programs inside the virtual machine. The start time of a freshly installed virtual machine is usually faster than that of an aged system, because no additional drivers and programs are installed. As seen in Table 2 the start time of the guest OS is only 50% of the host OS. A minimum install of Windows XP usually boots in 30 seconds, but antivirus and network drivers delay such fast boot times. The minimum memory requirements are quite astounding with 150 MBytes but the real-time antivirus software needs an additional 50 MBytes. In comparison a 3 year old production system needs 750 MByte, with additional restrictions that 32-bit Windows systems can only allocate and use 2.8 GBytes even if more memory is installed. The problem is that many programs and hardware driver software for mass spectrometers are not yet certified for 64-bit operation and would create incompatibilities. One solution here is to install a large memory system with a 64-bit operating system as host OS and use the 32-bit machines as guest OS, allowing both 32-bit and 64-bit operation. Windows 64-bit can directly emulate 32-bit programs in an emulation layer. If, however, 64-bit drivers are required and not yet available the program can not be installed in the first place.

### Discussion of cheminformatics and mass spectrometry related benchmarks

The single core benchmarks NIST SciMark 2.0a, Molgen Demo, CDK Descriptor GUI, Seven Golden Rules are all single-threaded benchmarks. Table 2 shows that the speed penalty within the virtual machine is around 5-10% for each of the programs. No investigation of the impact of disk speed was performed. But the fast Areca RAID-6 system allows full guest CPU utilization. Therefore the penalty on disk use exists but is very small on a hardware RAID system. In case of a slow single hard disk on the host system, the disk performance within the guest system is also lower. In such a case the disk system overhead from guest and host system add up and decrease the overall disk speed. Many new desktop computer systems utilize minimum two CPUs. The new Intel Nehalem Core i7 technology provides fast quad-core CPUs each with a total of eight working threads. Unfortunately only few chemistry and mass spectrometry desktop applications are multi-threaded or multi-core ready [37]. Among the tested versions which can make use of multi-core systems are the JChem calculational routines and MZMine2 for chromatographic alignment. The speed penalty on single-threaded programs compared to a dual core setup is severe. The stereoisomer calculation shows that on a dual processor machine a doubled performance can be obtained. Unfortunately, the Microsoft Virtual PC is a single-threaded application and does not allow the use of multi-core CPUs in the guest virtual environment. Ironically, when Microsoft bought the Virtual PC technology from Connectix in 2003 the software supported symmetric multi-processing (SMP) virtual machines. The free Microsoft Virtual PC 2007 is marketed as a desktop virtualization product and the free Microsoft Virtual Server 2 is marketed as a server product and can utilize multi-core CPUs. In comparison, the commercial VMWare Workstation and the open source VirtualBox both support virtual symmetric multiprocessing (SMP) and currently up to 32 virtual CPUs can be used in the guest system. The MZMine2 test especially shows the disk I/O dependence because of the large file size and the multi-core CPU dependence, because the software can be executed according to the number of available threads on the computer and therefore performs with double speed on a dual CPU setup.

### Virtual machines can diversify operating system choices in chemistry labs

The majority of software that is commercially sold together with mass spectrometers is running under Microsoft Windows. One reason may be the sole availability of Microsoft Windows driver software for analog-digital converters (AD/DA) which are required when connecting mass spectrometers to PCs. However there is no explanation why LINUX installations cannot be used because many older instruments were successfully running under different UNIX operating systems. The reason of developing vendor software only for a single operating system is based on the complexity of the software development tools and the development and support costs. Aiming at a single platform certainly reduces costs for the vendor. Hardware near programming furthermore usually requires C or C++ code development. The data evaluation part can be done on multiple platforms including Linux, Mac OS and Windows. Here cross-platform applications written in JAVA, which have the ability of running on many different operating systems have a clear advantage.

Modern mass spectrometry labs usually use multiple operating systems for historic reasons. Windows computers are used for operating chromatography equipment and mass spectrometers, LINUX OS for running software on computer clusters and Mac OS X for personal workstations and laptops. However only very few mass spectrometry desktop applications are available for MacOS [38]. That problem can be solved by using a virtual PC application like VMware Fusion or Parallels Desktop for MAC to install Windows compatible applications. As already mentioned it is currently prohibited by Apple Inc. to run Mac OS X as a guest operating system in a virtual machine on non-native apple hardware.

The choice between 32-bit and 64-bit operating systems [39] for mass spectrometry based computer systems is based on two major factors: If the computer has more than 4 GByte RAM available and the motherboard and CPU are 64-bit capable it is recommended to use a 64-bit operating system to utilize more than 4 GByte memory. In case of less than 4 GByte RAM a 32-bit system is sufficient. The other major obstacle is the availability of programs and drivers that are natively compiled for 64-bits. If such 64-bit software drivers are not available for hardware cards (AD/DA converters, PCI cards) then it is impossible to use that hardware on a 64-bit operating system. In case of 32-bit software this is not a major problem, because most 64-bit operating systems can execute both 32-bit and 64-bit software. It is recommended however to test all system critical software on a virtual machine before deploying them in a working environment.

### Hardware choices for software virtualization in chemistry labs

For server consolidation purposes usually server-grade components are used. That includes a motherboard capable of multi-socket CPU setups and enough memory banks to handle memory from 32 to 512 GByte RAM. As CPUs the quad-core or hex-core Intel XEON (based on Nehalem technology) as well as AMD Opteron (based on Shanghai or Istanbul 45 nm technology) can be recommended. The overall hard disk performance is extremely important for virtualization, therefore a series of 10,000 rpm SAS or SATA hard drives using RAID6 or RAID10 hardware RAID controllers (such as ARECA, LSI, ADAPTEC, 3WARE) should be used. A native hypervisor virtual machine monitor (XEN, VMware ESX Server or Microsoft Hyper-V) can be installed as core software layer. Any LINUX or WINDOWS guest operating system can be installed into the hypervisor.

For desktop virtualization a dual-core or quad-core processor (Intel Core i7 or AMD Phenom) should be used. The memory can range from 2 to 32 GByte. As operating system any 64-bit LINUX, MAC or WINDOWS system can be installed. For each virtual machine a minimum of 800 MByte RAM should be considered. Therefore if the host operating system uses 1 GByte RAM and it is planned to run four virtual machines in parallel, a minimum of 4 GByte RAM is needed. As hard disk system an Intel Matrix software RAID with multiple disks can be used. For even higher performance a Solid State Disk (SSD) setup or server grade hard disks with an ARECA RAID controller are recommended. Currently only limited support for Direct3D graphics cards and other specialized hardware inside virtual machines are provided. Applications that require such hardware should be run on native systems. Figure 5 shows an Intel Nehalem 3 GHz quad-core system equipped with four hard disks in RAID10 (mirrored stripe). The system can be used to virtualize special hardware, like a 32 thread LINUX machine as seen in the screenshot. The high average hard disk transfer rate of around 200 MByte/sec is important because the system has to deal with multiple virtual machines all performing their own disk operations. The most common source of mediocre performance of virtual machines is the use of a single slow hard disk.

### Software licensing issues using virtual machines

Each operating system installation requires its own operating system license. In case of the free desktop LINUX operating system no licensing issues occur. In case of Microsoft Windows or LINUX Enterprise versions a separate license for each computer processor and for each virtual machine install must be acquired. That is also the case for most commercial software if not otherwise stated in the EULA. For universities, academic volume licenses are usually available at a reduced price. In case of trial licenses, a use in production environments is mostly prohibited. For teaching environments a special agreement must be reached with the software vendor. Some companies like ChemAxon provide three different kinds of license a) paid commercial licenses b) free teaching licenses and c) free academic licenses for use in an academic research environment.

### Server consolidation in research labs using virtual machines

The use of server consolidation approaches is very common in larger research laboratories or bioinformatics labs at universities. Figure 4 (right side) shows a XEN virtual machine monitor running multiple VMs at the UC Davis Genome Center Bioinformatics core lab. The idea is that a single physical computer with multiple CPUs and large memory setups runs different operating system and provides multiple services at once. That can include different web services, database front-ends or web sites. Additionally internal computations can be carried out on such a system and the system can also be used to provide intermediary backup solutions. With connected small diskless network PCs such a system could even provide simple common services as Word, EXCEL, PowerPoint and access to statistical services, without the need of purchasing individual computers for each student and researcher. Larger virtualization projects including several thousand virtual machines are usually deployed by computer IT departments at large research universities. The aims are the same: minimizing management and hardware costs.

### Use of virtual machines for software testing and distribution and computer upgrades

A common application of virtual environments is application testing and development. Especially computer programmers use such VM technologies for testing the deployment of their software for cross-platform use under different operating systems. The distribution of existing (open) software packages is usually performed with live-CDs that contain a series of programs on a bootable LINUX CD [40]. Especially the Bioinformatics community has a strong history of using live-CDs such as the VLinux and the Vigyaancd for software distribution [41]. Such CDs can be converted to a single ISO file that can be mounted inside a virtual machine, allowing the LINUX system to run without the need to reboot. Although the direct distribution of pre-installed virtual machines is widely used in the computer science community the use in chemistry is very sparse. Some examples include the ECCE (Extensible Computational Chemistry Environment) [42] or the MASPECTRAS platform for management and analysis of proteomics data [43]. Complex installation processes and web server installations on production systems can be easily avoided by using such pre-configured virtual hard disks (VHDs). That also includes applications for grid computing inside virtual machines [14, 44]. It is must be realized that Windows software inside a pre-configured Windows system is not suited for worldwide distribution or outside a university with volume licensing, because each installation requires a paid license. The use of the WINE emulator [6] that is capable of running Windows software inside a LINUX virtual machine could be a possible solution.

Another application for VMs is legacy software testing. Certain programs may only run under native 32-bit environments. Although Windows has an in built 32-bit legacy emulation, the only solution may be to install a 32-bit and a 64-bit OS into an virtual machine. Older native Windows 16-bit installer programs will not run on Windows 64-bit computers, making the use of a 16-bit or 32-bit virtual machine a first choice. Also software which requires older operating system versions can be tested without problems. Once an application is installed and shows erroneous behavior or major incompatibilities a snap-shot from an older date can be used to restore the virtual machine to its original condition. Another favorable use is to replace old computer infrastructure but retain or keep all the current software installations. In such a case a full copy of the hard disk is created and this virtual hard disk file (VHD) is installed into a virtual machine on a faster and newer computer. Popular software tools for such a purpose are the freely available VMWare Converter and the Microsoft Disk2vhd software. In a teaching environment the use of virtual PCs is useful if no hardware based hard disk write protection is in use. In such a case the virtual machine installations including all intentional and unintentional changes can be discarded after each class and for a new teaching class a fresh original copy is simply restored.

### Use of virtual machines for better computer safety - avoiding viruses, Trojan horses, zombie farms and drive-by-infections

Computer safety in chemistry and life sciences research labs not only includes anti-virus scanners and multiple stage backups of important scientific data but requires also a more active approach towards virus prevention. Current anti-virus software can detect more than 70,000 threats and viruses. However that requires the virus to be known to the anti-virus software. Any potential new virus or software exploit cannot be detected, leaving a critical exploitation time window open until virus updates are provided. Trojan horse programs can be installed to either steal passwords or log every keyboard keystroke or deactivate internal software firewalls. Such compromised systems are used from outside as servers or proxies for illegal material including music files, video files, commercial software or pornographic material. Especially proxy functions are dangerous because a computer outside a research organization or university can now access files or network ranges that are usually only accessible for computers inside the organization. This is due to the fact that many authentication schemes work IP network address based, hence assuming the computer is a registered and clean system belonging to the internal network. Unsecured and unpatched computers can become parts of large botnets which in case of large botnets like Conficker or Torpig have infected hundreds of thousands of Windows PCs [45]. The infected PCs (zombies) are controlled by Zombie Masters which use such computers to extort money, gain information, steal credit card data or rent subnets to persons that want to perform DDOS (Distributed Denial of Service attacks) or send spam mail.

The use of LINUX or Mac OS systems can actually prevent virus spread, but not because such systems are generally safer, but due to the fact that administrator rights are handled very strict. LINUX computers are also prone to attacks, a recent severe vulnerability of LINUX systems in August 2008 allowed the Phalanx2 kernel rootkit to be installed and steal SSH passwords and subsequently get access to other systems [46]. One of the bigger problems in terms of computer safety is that many older programs under Windows XP always require administrator rights for installation and switching back to more restrictive user rights will let the program fail. Applying only guest user rights and installing all required software updates already reduces the number of possible virus attacks.

Research centers and universities are commonly protected by multiple hardware firewalls or have internal safe-zones without any internet access or even computers prohibiting any data exchange. Under realistic scenarios such extreme protection without any internet access is contra-productive. Even with hardware firewalls activated, users are allowed to surf the internet (IP port 80) or allowed to use SSH and SFTP (IP port 21) for connecting to remote computers or compute clusters. In such a scenario virus or Trojan horse infections can still occur. Another solution would be to surf the internet through proxy software that monitors all incoming traffic with multiple anti-viruses and rootkit detection utilities. Such a software solution which includes web antimalware, and https (secure traffic) and http web traffic inspection, a network inspection system and URL filtering is available for enterprise customers [47]. A simpler solution would be to use online surfing tools like SiteAdvisor [48] or simply use Sandbox technologies [49] as implemented in the Google Chrome Browser which restricts program rights.

The use of virtual machines for internet connections and surfing is highly recommended. If a user works on a Windows Machine as host and surfs the Internet using a small LINUX OS as host he would greatly reduce direct computer virus infection risks. Usually the computer virus itself cannot escape the virtual environment, therefore the underlying operating system and programs remain virus free. It must be mentioned that programs can detect if they are executed inside a virtual environment [50] and that there are few concept studies of ultrathin hypervisors (Blue Pill/Red Pill) which can be exploited as rootkits [51]. The second barrier would be the cross-platform barrier because only few viruses exist which could be executed in LINUX and Windows together. Such a scenario requires that the guest operating system itself is not prone to any network attack from outside. The Windows Operating System 7 has inbuilt virtual machines services, therefore browser sessions could be automatically started within a virtual machine. Using sandbox browsers (Google Chrome) already reduces the risks of virus infections, but such natively running browsers are still prone to vulnerabilities from installed external plugins (Flash, PDF, bitmaps graphics, QuickTime). Most viruses can not escape from a virtualized cross-platform environment and the virtual image itself can be reset to the original stage, therefore preventing any virus infection.

### Use of virtual machines in teaching environments

Cheminformatics and mass spectrometry teaching not only require the classical chalkboard talks but also laboratory sessions. In such experimental laboratory classes students perform experiments on different mass spectrometry platforms including time of flight analyzers (TOF), Orbitrap analyzers, Fourier-transform mass spectrometers and investigate different ionization modes including electrospray, matrix-assisted laser desorption ionization (MALDI) and others. Such experimental classes have to be taught in smaller group sizes, because not all students can perform the experiment itself, but they should be involved as much as possible. An estimated 80% of the time will be spent on software work and data evaluation of acquired spectra and the investigation of mass spectra and their associated molecular structures. Therefore, a strong cheminformatics syllabus and software handling courses are needed. We observed in our class that prior to the course students were mainly exposed to internet search, Microsoft Word and EXCEL and general purpose chemistry drawing programs. Very few individuals had computer programming skills. Individual discussions revealed that the software classes had direct synergistic impacts, with students independently exploring the learned databases and chemistry programs.

**Figure 6:**
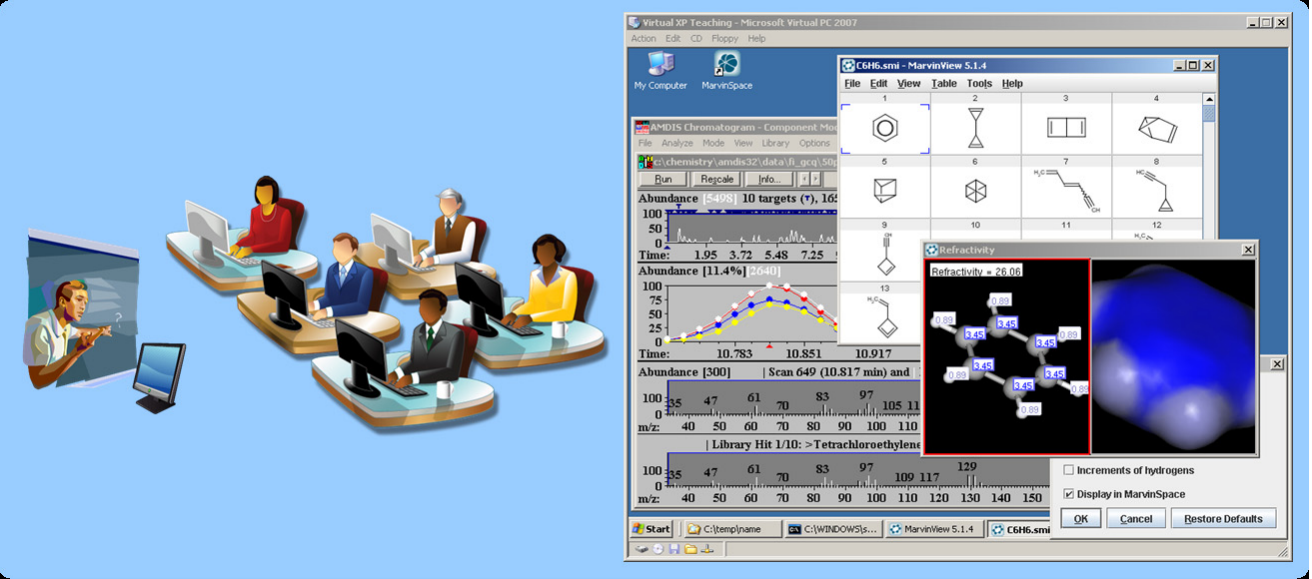
**Hands-on labs: Virtual machines are used for teaching cheminformatics and mass spectrometry software classes**. The hands-on class provides everybody with the same software and setups hence avoids installation and settings problems. All required software is installed and tested on a single virtual machine and this software image is later deployed to all computers in the class room. Right picture: Screenshot of the teaching VM with WIN XP and the AMDIS and MarvinView software running.

For theoretical teaching distance learning techniques [52, 53], platform-independent chemistry web services [54–56] or podcasting techniques of lectures in video and audio MP3 are [57] commonly used and well accepted. For direct on-site teaching of computer applications and approaches the use of virtual machines is recommended (see Figure 6). Such hands-on classes provide real world experience with software programs. Tasks that are commonly performed in the laboratory can be tested in a non-destructive virtual environment. In case of user input errors, user modifications or program crashes, the virtual disk image can be easily used to restore the original virtual machine. An additional advantage is that everybody uses the same software settings and setups, hence installation and settings problems are avoided. If only open-source or free software installations are used it would be possible to freely distribute such pre-configured virtual machines for cheminformatics teaching to a broad community.

A possible solution for cheminformatics distance learning, based on remote access to virtual machines, would be the use of a large server and a virtual machine setup with around 48 CPU cores and 256 GByte RAM. Students would then login via remote desktops or thin clients into virtual machines with teaching material. Existing software technology allows access to such services via internet browsers JAVA or ActiveX plug-ins. However in the current computer teaching laboratory setup 42 workstations with overall 82 cores and 164 GByte of RAM were available. In case of a virtual machine setup not only a powerful but expensive server had to be purchased but also individual diskless thin client computers and additional software licenses had to be acquired. Due to the optimized system management the use of individual computers in the lab was the preferred and overall cheaper solution. However, for distance based learning a total virtualization could be a good solution to reduce administration time.

### About the missing cheminformatics education at universities worldwide

A general problem is, that compared to bioinformatics, cheminformatics is taught only at few universities [58] with strong cheminformatics graduate-level and PhD programs. Related courses are sometimes taught together with computational chemistry, quantum chemistry or theoretical chemistry courses. Among those few universities are Indiana University, UC Irvine, Clarkson University, University of Michigan, University of New Mexico, Louis Pasteur University of Strasbourg (France), University Erlangen Nuremberg (Germany), Beilstein-Stiftungsprofessur Chemieinformatik at the University Frankfurt/Main (Germany) and University of Sheffield (UK) [59] and New University of Lisbon (Portugal) [60].

**Figure 7:**
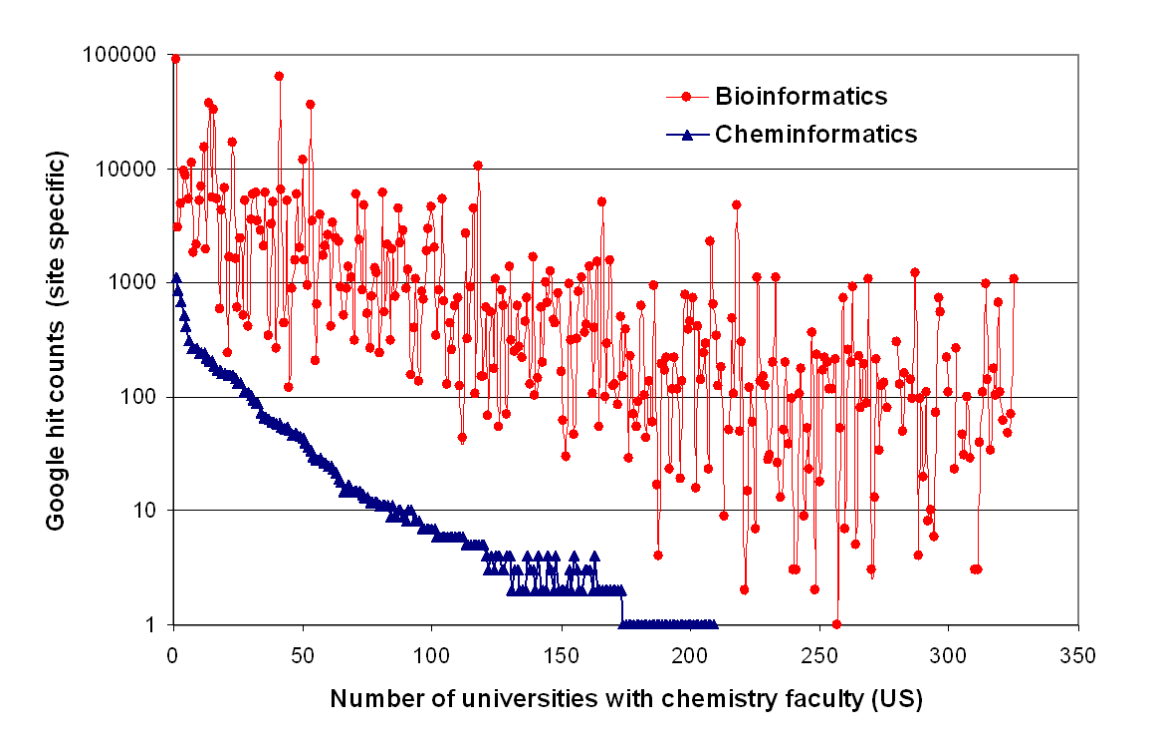
**Popularity of cheminformatics vs. bioinformatics based on site specific Google hit counts across 325 universities (US) with research chemistry faculty**. For all 325 universities a site specific search on Google was performed and mapped on the graph, i.e. *cheminformatics site:berkeley.edu* returned 93 hits and *bioinformatics site:berkeley.edu* returned 3620 hits. Because UC Berkeley hosts more bioinformatics related material it is safe to assume that bioinformatics is more popular than cheminformatics at UC Berkeley. Around 100 universities had no occurrence of the words *cheminformatics* or *chemoinformatics* on their global university websites (scores combined); Search date: August 2009.

Cheminformatics is certainly related to chemometrics and computational chemistry [61] but all three sciences cover specialized areas of chemistry with large overlaps. A comparison of the relative popularity (based on site specific Google hit counts) of cheminformatics versus bioinformatics among 325 chemistry universities and institutions across the US can be found in Figure 7. For all of the 325 universities with chemistry faculty a domain specific Google search was performed to obtain information how often the word "cheminformatics" or "bioinformatics" can be found across the whole university website. For example a Google search for *cheminformatics site:berkeley.edu* returned 93 hits and *bioinformatics site:berkeley.edu* returned 3620 hits. Therefore more bioinformatics related material (web pages, PDF documents) can be found at the UC Berkeley website. It can be concluded that bioinformatics is more popular than cheminformatics at UC Berkeley. The figure shows that the term *BioInformatics* is found almost two orders of magnitude more frequently than *ChemInformatics* or *ChemoInformatics* combined across all universities. The number of total hits for bioinformatics (315 institutes and 647082 total hits); cheminformatics (189 institutes and 5905 total hits) and for chemoinformatics (153 institutes and 4582 total hits) confirm the popularity of bioinformatics in modern life sciences.

The current fast-paced chemistry development requires that each chemistry student should have a very early cheminformatics education which goes beyond simple database search and structure drawing [62]. The use of pre-configured virtual machines containing teaching material and cheminformatics software could lead to an easier handling of complex software setups. Also distance based learning techniques could use real-time remote connections to virtual machines equipped with a wide array of cheminformatics software [63, 64]. Such advanced learning tasks could include in-silico reaction planning with a large computerized reaction database and planning system [65], the use of molecular descriptors, in-silico de-novo molecular design, structure screening and searching, Quantitative Structure-Activity Relationships (QSAR) and visualization methods [66]. Also the lack of programming skills among chemists must be regarded as a potential threat to a successful development of the field. The recruitment of computer scientist from outside the field is limited, because chemical structure handling and chemical reaction manipulation requires a deep understanding of chemistry in the first place. Wendy Warr an international expert in chemical information stated in a 2008 editorial [67]: "*The catalog of courses and resources compiled in this paper might suggest that cheminformatics education is flourishing. It is not. Many examples of isolated efforts are cited here but there is no European or international coordination. Cheminformatics practitioners have still not defined their discipline and its impact, let alone successfully made a case to governments and funding agencies*."

In a related commentary about systems chemical biology [68], the authors discussed cheminformatics tools that can integrate chemical knowledge with biological databases and raised concerns about the cancellation of the National Institute of Health (NIH/US) funding projects for the "Preapplication for Cheminformatics Research Centers" in 2007 [69], which would have been the largest funding source for the study of new cheminformatics approaches in the United States. It must be argued that cheminformatics education and research are such a fundamental part of *new chemistry*, that funding in the United States should be provided by the National Science Foundation (NSF) and not by the NIH which historically receives a much higher funding (Budget FY2009 NIH: 30.5 billion US$ and NSF: 6.5 billion US$ [70]). The NSF not only has the mandate of promoting interdisciplinary research but also has a strong interest in chemistry education [71]. Regrettably, U.S. funding of chemistry can barely keep up with inflation [72] and the FY2009 budget for the NSF Division of Chemistry (CHE) is around $244.67 million and therefore represents only 3.7% of the whole NSF budget. But making the case that cheminformatics is a substantial building block for success in the grand challenges in chemistry [73] is up to the cheminformaticians themselves.

## Conclusion

Software virtualization in chemistry, mass spectrometry and cheminformatics is needed for software testing and development under different deployment scenarios and operating systems without the need of having multiple standalone computers. We have shown with multiple cheminformatics and mass spectrometry software benchmarks that the computational penalty of using virtual machines is very low and usually around 5% to 10%. In order to obtain maximum performance the virtualization software must be multi-core enabled and should emulate a multiprocessor configuration in the virtual machine environment. The computational chemistry software should make use of multi-core CPUs and the computer itself should be equipped with a multi-core CPU as well as a fast SSD or RAID system. Software virtualization in research chemistry labs is useful for keeping the computational infrastructure small and manageable. Multiple operating systems can be used one multi-core CPU computer providing web services, backup services, computational and data exchange services. Software virtualization in a teaching environment allows faster deployment and easy use of commercial and open source software. Preconfigured virtual machines can be used for worldwide distribution of open source and freely available cheminformatics tools.

## Electronic supplementary material

Additional file 1: **Popularity of cheminformatics vs. bioinformatics - complete statistics**. Listing of 325 US universities with chemistry program; Domain specific search and statistics(using the Google API web search) of the occurrences of the words: Cheminformatics, ChemoInformatics, BioInformatics, Chemometrics, Computational chemistry, Chemical Informatics; Format: Microsoft EXCEL 2003; Curator: Tobias Kind; FiehnLab August 2009; http://fiehnlab.ucdavis.edu/staff/kind/ (XLS 370 KB)

Below are the links to the authors’ original submitted files for images.

Authors’ original file for figure 1

Authors’ original file for figure 2

Authors’ original file for figure 3

Authors’ original file for figure 4

Authors’ original file for figure 5

Authors’ original file for figure 6

Authors’ original file for figure 7
