# Supplementary figures and images for: Software platform virtualization in chemistry research and university teaching
Source: J Cheminform. 2009 Nov 16;1:18. doi: 10.1186/1758-2946-1-18 (PMC2820496; doi:10.1186/1758-2946-1-18)

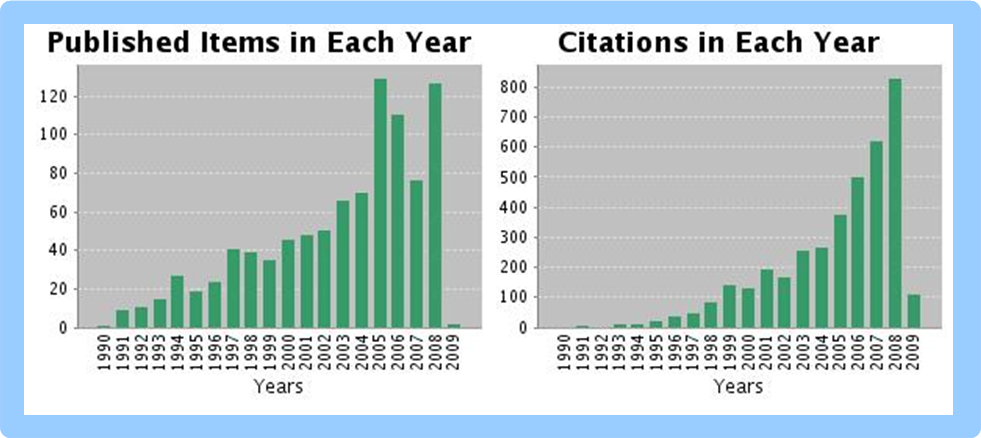

Supplement: Supplementary file 2 — Authors’ original file for figure 1 [file 13321_2009_18_MOESM2_ESM.tiff]

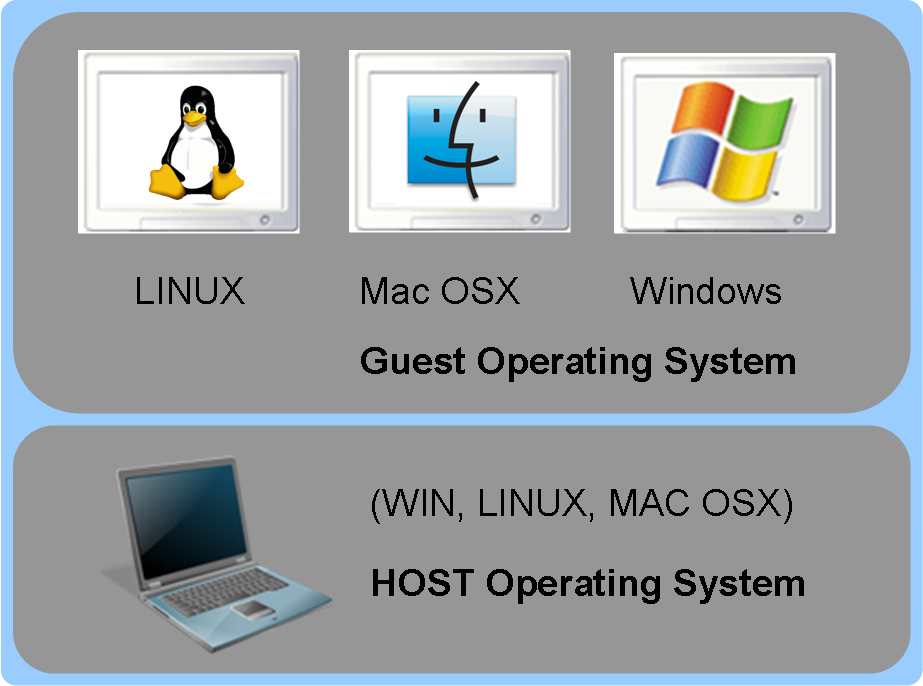

Supplement: Supplementary file 3 — Authors’ original file for figure 2 [file 13321_2009_18_MOESM3_ESM.tiff]

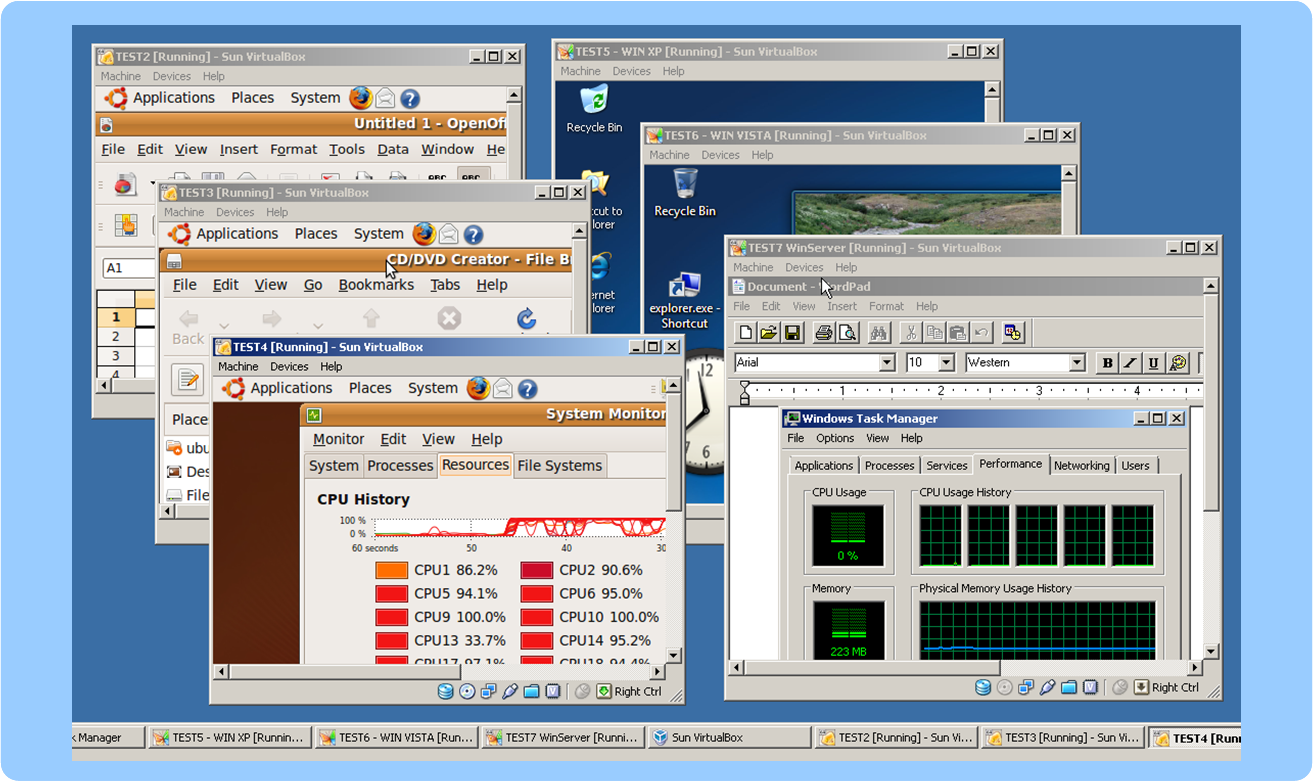

Supplement: Supplementary file 4 — Authors’ original file for figure 3 [file 13321_2009_18_MOESM4_ESM.tiff]

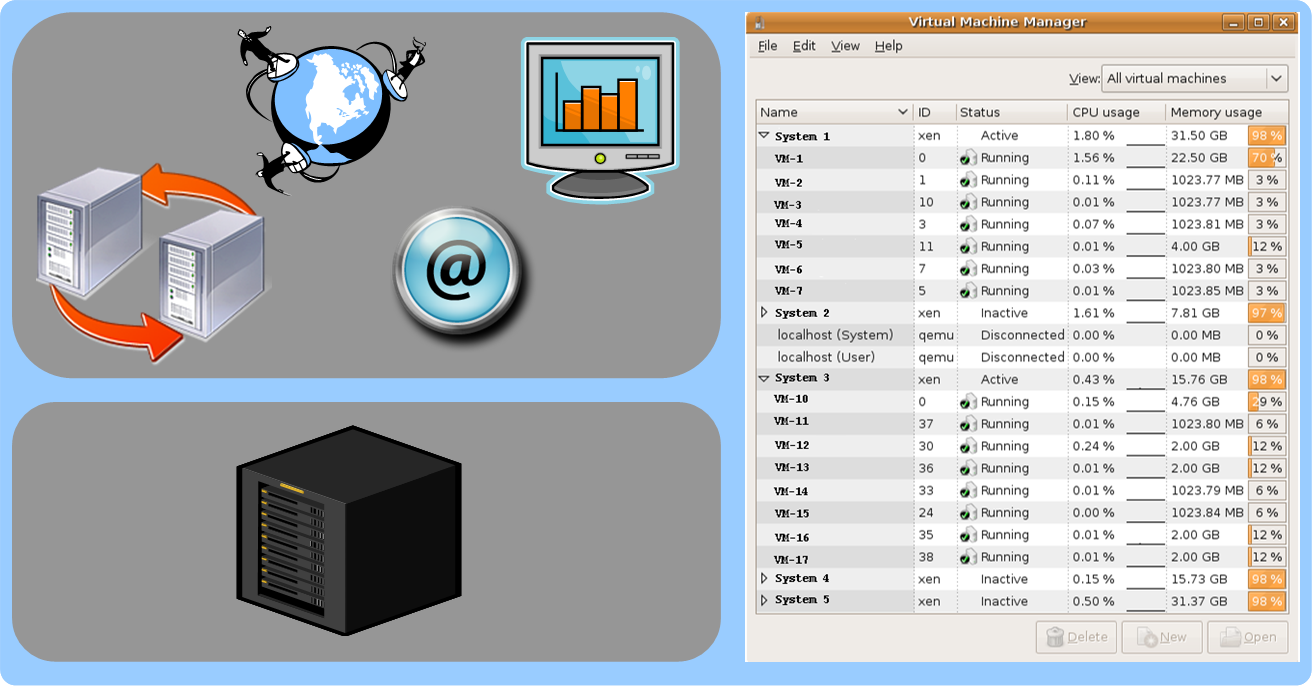

Supplement: Supplementary file 5 — Authors’ original file for figure 4 [file 13321_2009_18_MOESM5_ESM.tiff]

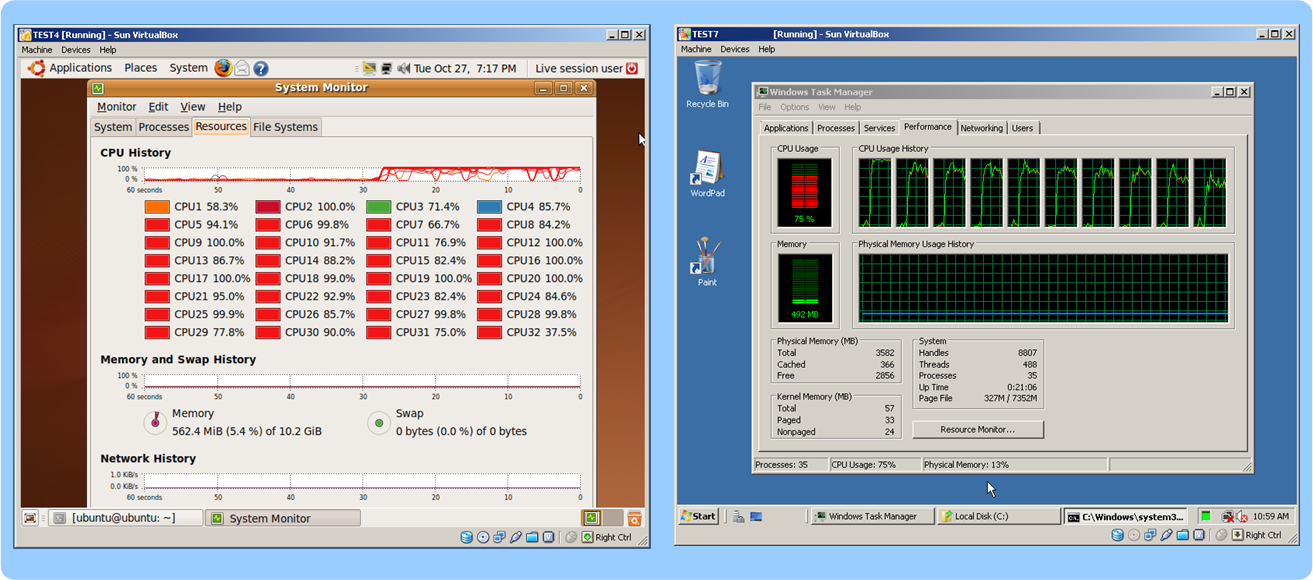

Supplement: Supplementary file 6 — Authors’ original file for figure 5 [file 13321_2009_18_MOESM6_ESM.tiff]

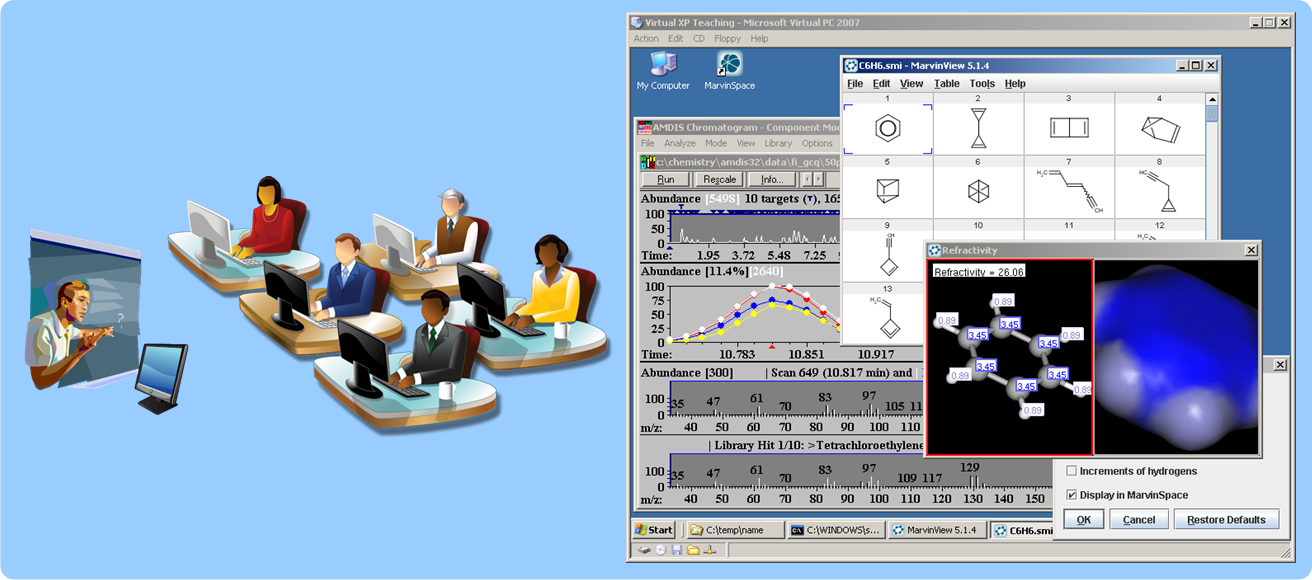

Supplement: Supplementary file 7 — Authors’ original file for figure 6 [file 13321_2009_18_MOESM7_ESM.tiff]

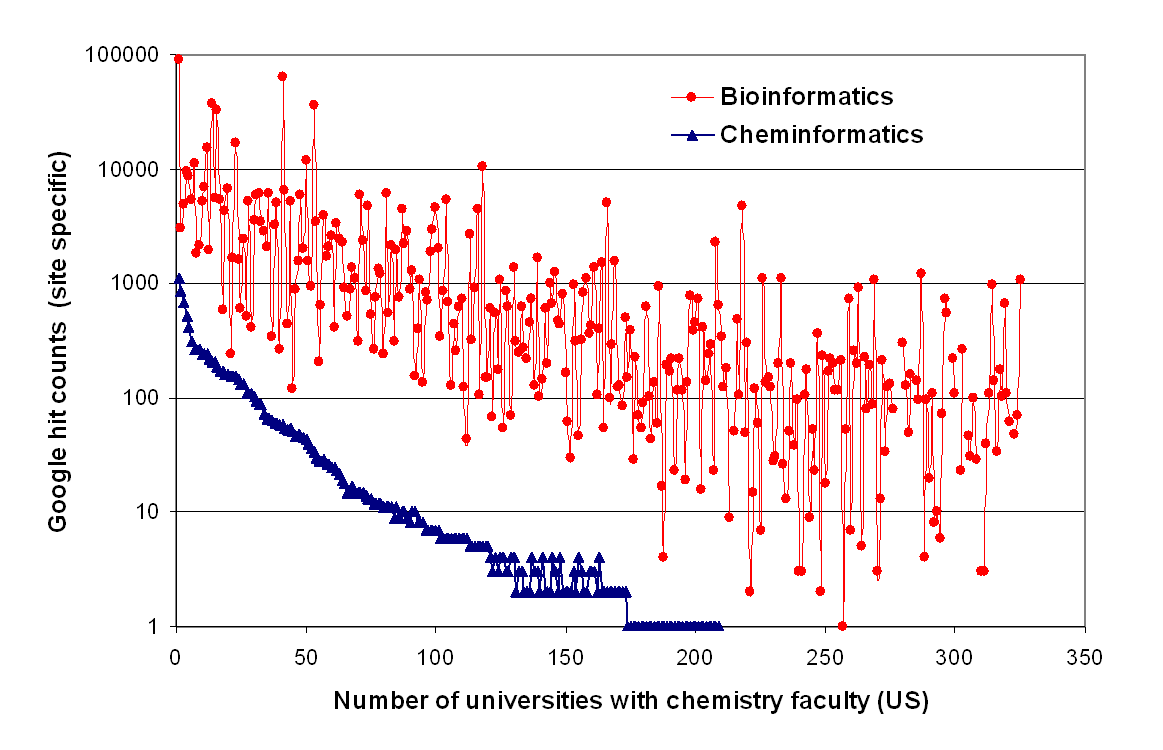

Supplement: Supplementary file 8 — Authors’ original file for figure 7 [file 13321_2009_18_MOESM8_ESM.png]
